# PadR-type repressors controlling production of a non-canonical FtsW/RodA homologue and other trans-membrane proteins

**DOI:** 10.1038/s41598-019-46347-w

**Published:** 2019-07-11

**Authors:** Samuel Hauf, Lars Möller, Stephan Fuchs, Sven Halbedel

**Affiliations:** 1 FG11 Division of Enteropathogenic bacteria and Legionella, Robert Koch Institute, Burgstrasse 37, 38855 Wernigerode, Germany; 2 ZBS 4 - Advanced Light and Electron Microscopy, Robert Koch Institute, Nordufer 20, 13353 Berlin, Germany; 3 FG13 Nosocomial Pathogens and Antibiotic Resistances, Robert Koch Institute, Burgstrasse 37, 38855 Wernigerode, Germany

**Keywords:** Bacterial genes, Pathogens, Food microbiology, Bacterial genetics

## Abstract

The Gram-positive bacterium *Listeria monocytogenes* occurs ubiquitously in the environment and infects humans upon ingestion. It encodes four PadR-like repressors, out of which LftR has been characterized previously and was shown to control gene expression in response to the antibiotic aurantimycin produced by other environmental bacteria. To better understand the PadR regulons of *L*. *monocytogenes*, we performed RNA-sequencing with mutants of the other three repressors LadR, LstR and Lmo0599. We show that LadR is primarily responsible for the regulation of the *mdrL* gene, encoding an efflux pump, while LstR and Lmo0599 mainly regulate their own operons. The *lstR* operon contains the *lmo0421* gene, encoding a homolog of the RodA/FtsW protein family. However, this protein does not possess such functionality, as we demonstrate here. The *lmo0599* operon contains two additional genes coding for the hypothetical trans-membrane proteins *lmo0600* and *lmo0601*. A striking phenotype of the *lmo0599* mutant is its impaired growth at refrigeration temperature. In light of these and other results we suggest that Lmo0599 should be renamed and propose LltR (listerial low temperature regulator) as its new designation. Based on the nature of the PadR target genes we assume that these repressors collectively respond to compounds acting on the cellular envelope.

## Introduction

The Gram-positive bacterium *Listeria monocytogenes* is the causative agent of listeriosis, which is one of the most serious foodborne bacterial infections in humans. The bacterium occurs ubiquitously in the environment and infects humans after consumption of contaminated food. Uncooked and ready-to-eat foods pose the highest risk of infection, the latter because *L*. *monocytogenes* is able to grow at refrigeration temperatures^1,2^. Fatality rates of listeriosis are remarkably high compared to other foodborne bacterial pathogens^3^, and, hence, the control of *L*. *monocytogenes* in food is of utmost importance for the food-processing industry. *L*. *monocytogenes* frequently enters the food chain due to its wide-spread presence in the soil, in surface waters, on plants and in the gut of various animals^4^. Aggravating this situation, the bacterium has a profound capacity to resist many conditions used to prevent food spoilage. It is not only able to grow at 4 °C, but it can also grow at high salt concentrations, accepts a wide pH range for growth and tolerates anti-microbial compounds of cold smoke^5,6^. *L*. *monocytogenes* readily forms biofilms on glass, plastic and steel surfaces^7^, often complicating effective disinfection of food-processing plants. Moreover, isolates of *L*. *monocytogenes* frequently are resistant against commonly used disinfectants such as benzalkonium chloride^8,9^ and such benzalkonium-resistant isolates have caused big outbreaks in the past^10,11^. Thus, an improved understanding of the ecology, survival strategies and stress responses of *L*. *monocytogenes* is important to reduce the entry and of the bacterium into the food chain and its persistence in food-processing plants.

Among the diverse molecular mechanisms employed by bacteria to sense and respond to environmental stresses are the PadR-type transcriptional regulators. The eponymous protein for this class of repressors is the phenolic acid decarboxylase repressor PadR of the firmicute bacterium *Pediococcus pentosaceus*^12^. It activates the expression of phenolic acid decarboxylase (PadA) in response to the exposure to toxic phenolic acids. PadA then converts the toxic phenolic acids into less toxic products, thereby conveying resistance to high levels of phenolic acids^12,13^. Another well-studied PadR-type repressor, LmrR from *Lactococcus lactis*, activates expression of the *lmrCD* multi drug efflux pump genes upon exposure to small toxic compounds like the antibiotic daunorubicin^14,15^. Under non-inducing conditions, LmrR represses *lmrCD* transcription by blocking the P_*lmrCD*_ promoter^15,16^. Binding of effector molecules induces conformational changes in LmrR causing relieve of repression^14,17,18^ and compound excretion through LmrCD^19^.

In *L*. *monocytogenes*, the PadR-type repressor LftR controls the expression of the *lieAB* genes encoding another antibiotic efflux pump^20^. Recently, we demonstrated that this efflux pump is expressed when *L*. *monocytogenes* comes into contact with aurantimycin A^21^, a depsipeptide antibiotic with very potent bactericidal activity against Gram-positive bacteria and produced by soil-dwelling *Streptomyces aurantiacus*^22^. Thus, LftR and LieAB likely promote survival of *L*. *monocytogenes* when it comes in contact with *S*. *aurantiacus* in the soil, its natural reservoir^21^, from where it may eventually enter the food chain.

Next to LftR, three additional PadR-like repressors are encoded in the *L*. *monocytogenes* EGD-e genome: LadR, LstR and Lmo0599. LadR controls production of the multi drug resistance pump MdrL^23^, associated with benzalkonium chloride resistance^24,25^. LstR was linked to heat resistance^26^ and Lmo0599 has not been characterized until now. In order to better understand the function of PadR-like regulators in *L*. *monocytogenes*, we here have identified the regulons of the three mentioned repressors by RNA-Seq and studied the function of their primary effector genes in genetic and functional experiments. In light of the results obtained we suggest to rename Lmo0599 as LltR (listerial low temperature regulator).

## Results

### Identification of the LadR, LstR and LltR regulons

In order to determine the target genes of LadR, LstR and LltR, the *ladR* and *lstR* genes were removed from the genome by allelic exchange and *lltR* was replaced with a non-functional copy carrying the L49A R51A L52A triple mutation in its helix-turn-helix motif. This triple mutation (lltR*) was designed to prevent binding of LltR to its recognition site. Next, transcriptomes of the *L*. *monocytogenes* wild type strain EGD-e and its isogenic Δ*ladR*, Δ*lstR* and lltR* mutants were analyzed by RNA sequencing. This revealed massive (~150-fold) derepression of the *mdrL* gene in cells lacking LadR (Table 1), which is in good agreement with previous work demonstrating that *mdrL* expression is repressed by LadR^23^. MdrL is a multidrug efflux transporter and supposedly transports compounds like ethidium bromide, cefotaxime and other antibiotics out of the cell^27^. Moderate induction of the *lmo1618-1617* operon^28^ was also observed in the Δ*ladR* mutant (Table 1), as described previously^29^. This bicistronic transcription unit comprises the *lmo1618* gene coding for a MarR-type regulator followed by *lmo1617* encoding the multidrug resistance transporter MdrM. MdrM is involved in the secretion of the signal molecule cyclic diadenosine monophosphate (c-di-AMP), which activates the host cytosolic surveillance pathway during intracellular passages^29,30^. The expression of other genes was not affected, indicating that LadR specifically regulates the expression its three target genes, among which *mdrL* appears to be the primary one.

**Table 1:** *L*. *monocytogenes* genes deregulated in the Δ*ladR*, Δ*lstR* and lltR* mutants.

locus	function	fold induction Δ*ladR* /wt	P value
**upregulated in Δ** ***ladR***			
*lmo1409*	MdrL major facilitator superfamily efflux pump	153.8 ± 44.3	0.0003
*lmo1618*	MarR family transcriptional regulator	4.3 ± 0.4	0.0003
*lmo1617*	MdrM multidrug transporter	2.6 ± 0.8	0.0094
**upregulated in Δ** ***lstR***		**fold induction Δ** ***lstR*** **/wt**	
*lmo0421*	RodA-like rod shape-determining protein	145.0 ± 11.3	2.6 × 10^−5^
*lmo0423*	RNA polymerase factor sigma C	139.3 ± 19.3	8.6 × 10^−7^
*lmo0420*	hypothetical protein, HAD family hydrolase	54.9 ± 10.2	0.0001
*lmo0419*	hypothetical protein	7.1 ± 1.5	0.0035
*lmo2773*	putative transcription antiterminator	2.4 ± 0.5	0.0035
*lmo2050*	excinuclease ABC subunit A	2.3 ± 0.4	0.0037
**downregulated in Δ** ***lstR***			
*lmo1597*	hypothetical protein	0.5 ± 0.04	0.0083
*lmo0416*	putative transcriptional regulator	0.5 ± 0.01	0.0017
*lmo0417*	hypothetical protein	0.4 ± 0.02	0.0084
*lmo1839*	PyrP similar to uracil permease	0.2 ± 0.06	0.0074
**upregulated in** ***lltR*** *		**fold induction** ***lltR*** */wt	
*lmo0599*	LltR, PadR-like transcriptional repressor	150.6 ± 49.7	4.4 × 10^−05^
*lmo0600*	DUF1700 containing hypothetical protein	118.0 ± 34.3	0.0001
*lmo0601*	DUF4097 containing hypothetical protein	106.8 ± 23.8	4.8 × 10^−5^
*lmo0602*	hypothetical protein, N-acetyltransferase domain	12.7 ± 9.1	0.0011
*lmo0954*	LiaI phage shock protein	3.8 ± 1.1	0.0024
*lmo2487*	DUF4097 containing hypothetical protein	3.1 ± 0.7	0.0072
*lmo0955*	LiaH phage shock protein	2.6 ± 0.6	0.0027
*lmo1637*	putative ABC transporter, permease protein	2.5 ± 0.2	0.0003
*lmo0047*	putative lipoprotein	2.4 ± 0.4	0.0029
*lmo1636*	putative ABC transporter, ATP binding protein	2.4 ± 0.4	0.0043

Deletion of *lstR* caused strong (~140-fold) overexpression of the *lmo0421* and *lmo0423* genes (Table 1), which form a tricistronic operon together with *lstR* (*lmo0423-lstR-lmo0421*)^28^. While the *lmo0421* gene is one of six *L*. *monocytogenes* RodA/FtsW paralogs, it is unclear whether it is also involved in peptidoglycan chain polymerization^31,32^. The *lmo0423* gene shares homology with ECF-type sigma factors (27.9% identity to *Bacillus subtilis* σ^V^) and has been designated σ^C^ ^26^. All three genes of this operon have been implicated in heat stress response^26^ and σ^C^ is also important during cold adaptation^33^. Only few possible target genes are known for this sigma factor^34,35^ and its main target is the *lmo0423-lstR-lmo0421* operon itself  ^26^. The two divergently transcribed and uncharacterized *lmo0420* and *lmo0419* genes located downstream of the *sigC* operon are also considerably overexpressed in the Δ*lstR* mutant (Table 1). Transcriptional read-through could be an explanation for this. The remaining LstR-affected genes, among which is the *lmo0416* gene coding for a putative transcriptional regulator, show lower fold changes and it remains unclear whether these are direct or secondary effects.

A similar hierarchy in fold changes was observed for genes de-repressed in the lltR* mutant. Here, the *lltR-lmo0600-lmo0601* operon^28^ was massively overexpressed (~100- to 150-fold), most likely due to relief of auto-repression through LltR. Derepression of *lmo0602*, located downstream of this operon and transcribed in the same direction could be due to transcriptional read-through. Besides this, six mildly overexpressed genes were found, among those the *liaIH* operon, encoding phage shock proteins^36^, as well as the *lmo1636* and *lmo1637* genes coding for components of a putative ABC transporter (Table 1).

### Promoter fragments controlled by LadR, LstR and Lm0599

**Figure 1:**
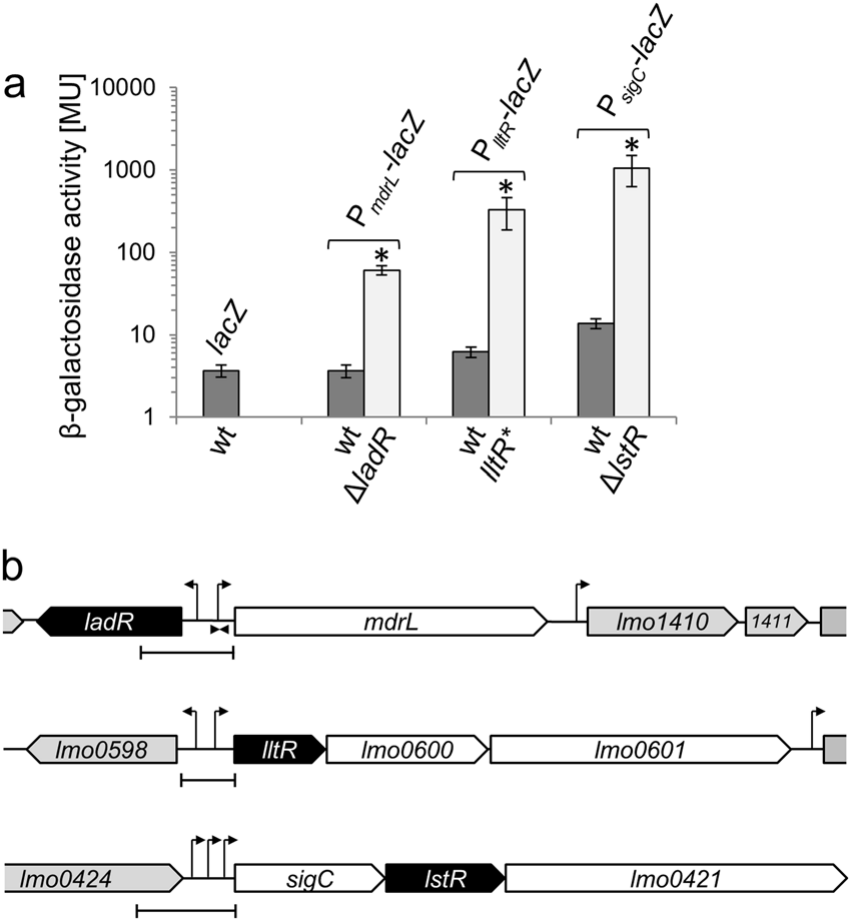
Activity of LadR-, LstR- and LltR-dependent promoters. (**a**) β-galactosidase activity in strains carrying *lacZ* fusions of the P_*mdrL*_, P_*lltR*_ and P_*sigC*_ promoters. Strains LMSH10 (P_*mdrL*_*-lacZ*), LMSH11 (Δ*ladR* P_*mdrL*_*-lacZ*), LMSH14 (P_*lltR*_*-lacZ*), LMSH15 (lltR* P_*lltR*_*-lacZ*), LMSH12 (P_*sigC*_*-lacZ*) and LMSH13 (Δ*lstR* P_*sigC*_*-lacZ*) were grown in BHI broth at 37 °C to mid-logarithmic growth phase and β-galactosidase activity was determined. The experiment was repeated three times and average values and standard deviations are shown. Asterisks indicate significant differences (*P* < 0.05). (**b**) Scheme illustrating gene arrangement at the *ladR*, *lstR* and *lltR* loci. Promoters are either adopted from experimental data^26,28^ or predicted using the bprom algorithm^56^. Promoter fragments used for construction of promoter-*lacZ* fusions are indicated.

To verify the data obtained by RNA-seq, fragments upstream of the *mdrL*, *sigC* and *lltR* genes, all between 200 and 370 bp in length and comprising the start codons of each gene, were fused in frame to the *lacZ* gene. These promoter-*lacZ* fusions were then introduced into wild type and mutant backgrounds lacking their cognate PadR-like repressors. Only background β-galactosidase activities, which did not exceed the values obtained with strain LMSH16 carrying a promoter-less *lacZ* gene for control, were observed for two of the three *lacZ* fusions in wild type cells. An exception was the P_*sigC*_ promoter that resulted in a roughly fourfold higher β-galactosidase activity in wild type cells when compared to the strain with the promoter-less *lacZ* (Fig. 1a). This increased background activity might be explained by the presence of three promoters in front of the *sigC-lstR-lmo0421* operon, including one σ^C^-dependent promoter^26^. Activity of the P_*sigC*_-promoter increased 77-fold in the absence of *lstR*, indicating that induced transcription of the *sigC-lstR-lmo0421* operon in the Δ*lstR* mutant is due to increased promoter activity. Likewise, β-galactosidase activity driven by the P_*mdrL*_ promoter increased in the absence of *ladR*, even though only 17-fold, and activity of the P_*lltR*_ promoter increased over 50-fold in the lltR* mutant (Fig. 1a). Taken together, the activities of the three tested promoters increase substantially in the absence of their cognate PadR repressor proteins. This indicates that LadR, LstR and LltR repress initiation of transcription from the P_*mdrL*_, P_*sigC*_ and P_*lltR*_ promoters, respectively. We tested induction of all three promoter-*lacZ* fusions in disc diffusion assays on X-Gal containing agar plates with rhodamine 6G, a known inducer of *mdrL* expression in *L*. *monocytogenes* LO28^23^, however, induction was not observed (data not shown). Likewise, none of the three promoter-*lacZ* fusions was induced by ethidium bromide, several antibiotics (penicillin G, fosfomycin, cycloserine, chloramphenicol, tetracycline, spectinomycin, ampicillin, kanamycin, nalidixic acid) or selected disinfectants (benzalkonium, tert-butylhydroquinone, acriflavine).

### Effect of PadR repressors on *in vitro* and *in vivo* growth of *L*. *monocytogenes*

**Figure 2:**
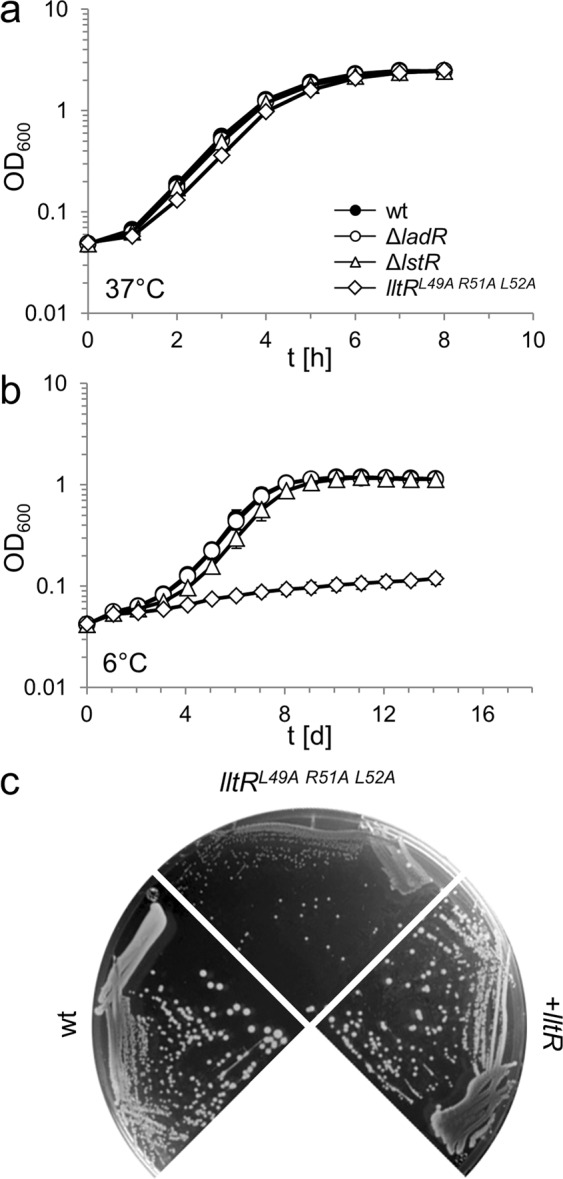
Growth of *L*. *monocytogenes ladR*, *lstR* and *lltR* mutants. (**a**) *L*. *monocytogenes* strains EGD-e (wt), LMSH1 (Δ*ladR*), LMSH2 (Δ*lstR*) and LMSH3 (*lltR*^*L49A R51A L52A*^) were grown in BHI broth at 37 °C. (**b**) Growth of the same set of strains at 6 °C. Growth curves were repeated three times and average values and standard deviations are shown. (**c**) Complementation of the cold-sensitive growth defect of the *lltR* mutant. *L*. *monocytogenes* strains EGD-e (wt), LMSH3 (*lltR*^*L49A R51A L52A*^) and the complemented strain LMSH42 (+*lltR*) were grown for six weeks on BHI agar containing 1 mM IPTG at 6 °C.

Next, we analyzed the contribution of the three PadR-like repressor proteins to growth in batch culture and inside eukaryotic cells. All three mutant strains grew like wildtype at 37 °C (Fig. 2a), 30 °C or 42 °C (data not shown). In contrast, strain LMSH3 carrying the mutated lltR* allele was nearly unable to grow at 6 °C, while the other two mutants showed wild type-like growth (Fig. 2b). This growth defect of the lltR* mutant was also evident during growth on BHI agar plates, but could be complemented by ectopic expression of a native *lltR* allele (Fig. 2c), demonstrating that the lltR* mutation and no secondary site mutation was the cause of the cold-sensitive phenotype.

The contribution of the three PadR-like repressors to intracellular growth was then studied using the J774 mouse macrophage infection model. This showed that the Δ*ladR*, Δ*lstR* and lltR* mutant strains were phagocytosed as the wild type strain and that their intracellular growth was unaffected (Fig. S1). Apparently, these proteins are not important during infection under the tested conditions.

### Identification of the effector gene of the *lltR* operon

**Figure 3:**
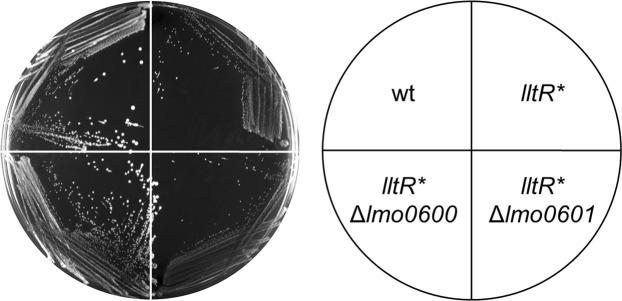
Identification of the effector gene of the *lltR* operon. *L*. *monocytogenes* strains EGD-e (wt), LMSH3 (lltR*), LMSH50 (lltR* Δ*lmo0600*) and LMSH51 (lltR* Δ*lmo0601*) were grown on BHI agar plates at 6 °C for six weeks.

In addition to the LltR repressor itself, the *lltR* operon encodes two more genes^28^. Immediately downstream of *lltR* is *lmo0600*, encoding a multi-spanning integral membrane protein of unknown function that contains a DUF1700 domain^37^. Further downstream there is *lmo0601* coding for a hypothetical exoprotein containing a DUF4097 domain^37^, which is annotated as a possible structural element of bacterial adhesins^38^. We wondered whether the cold-sensitive growth phenotype of the lltR* mutant results from overexpression of *lmo0600*, *lmo0601* or both genes. To study this, both genes were individually deleted in the lltR* mutant background and the ability of the resulting mutants to grow at 6 °C was then tested on BHI agar plates. This revealed that deletion of *lmo0600* restored normal growth in the lltR* Δ*lmo0600* double mutant, whereas the lltR* Δ*lmo0601* was as impaired to grow at 6 °C as the lltR* single mutant strain (Fig. 3). This shows that overexpression of the transmembrane protein Lmo0600 is detrimental for growth at low temperatures and emphasizes the importance of LltR in repression of *lmo0600* transcription for growth at refrigeration temperature.

### Analysis of the effector gene of the *sigC* operon

**Figure 4:**
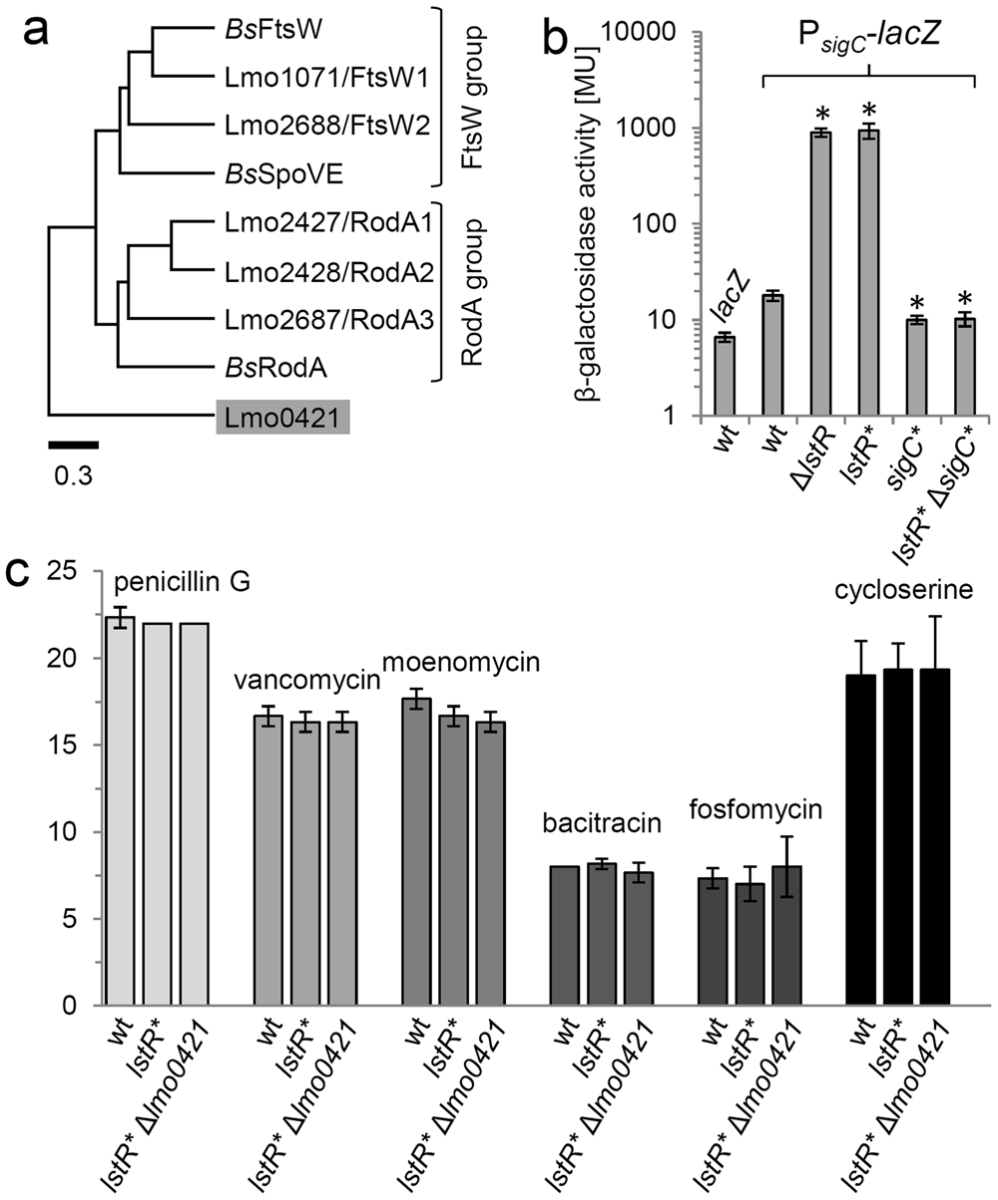
Susceptibility of *L*. *monocytogenes lstR* and *lmo0421* mutants against antibiotics targeting peptidoglycan biosynthesis. (**a**) UPGMA tree of the six *L*. *monocytogenes* and the three *B*. *subtilis* FtsW/RodA homologues. Legend: Substitutions per site. (**b**) Effect of the *lstR* ^*L90A L92A L93A*^ mutation and σ^C^ inactivation on the activity of the P_*sigC*_ promoter. *L*. *monocytogenes* strains LMSH16 (wt, *lacZ*) LMSH12 (wt, P_*sigC*_-*lacZ*), LMSH13 (Δ*lstR* P_*sigC*_-*lacZ*), LMSH63 (lstR* P_*sigC*_-*lacZ*), LMSH96 (sigC*; P_*sigC*_*-lacZ*) and LMSH97 (lstR* sigC* P_*sigC*_*-lacZ*) were grown in BHI broth at 37 °C to mid-logarithmic growth phase and β-galactosidase activity was determined. The experiment was repeated three times and average values and standard deviations are shown. Asterisks indicate significant differences (*t*-test, *P* < 0.01). (**c**) *L*. *monocytogenes* strains EGD-e (wt), LMSH39 carrying the *lstR*^*L90A L92A L93A*^ mutation (lstR*) and LMSH40 additionally lacking the *ftsW*/*rodA* homologue *lmo0421* (lstR* Δ*lmo0421*) were tested in filter disc susceptibility assays using penicillin G (1 mg/ml), vancomycin (20 mg/ml), moenomycin (1.6 mg/ml), bacitracin (40 mg/ml), fosfomycin (20 mg/ml) or cycloserine (30 mg/ml). Tests were repeated three times and average values and standard deviations are shown.

Of particular interest among the PadR-regulated genes is the third gene of the *sigC* operon, *lmo0421*, which represents the effector gene of this operon and encodes an uncharacterized protein with reasonable similarity to FtsW/RodA proteins. Recent evidence showed that these proteins constitute peptidoglycan glycosyltransferases^31,32^. *L*. *monocyctogenes* encodes six FtsW/RodA proteins in total^39^, and among these, Lmo0421 represents a non-canonical homologue that clusters separate from RodA and FtsW proteins (Fig. 4a). We wondered whether *lmo0421* could substitute for any of the two FtsW-like or any of the three RodA-like proteins of *L*. *monocytogenes* when overexpressed. To study this, we first constructed a novel *lstR* mutant (strain LMSH40), in which three critical amino acids in the conserved operator recognition site of LstR were replaced by alanines (*lstR* ^*L90A L92A L93A*^, designated lstR*). In this mutant, the overall architecture of the *sigC* operon remains intact, however the P_*sigC*_ promoter is de-repressed to the same degree as observed in a Δ*lstR* deletion mutant (Fig. 4b). Interestingly, expression of the *sigC* operon was completely dependent on σ^C^, as inactivation of the *sigC* gene in wild type and in lstR* backgrounds reduced activity of the P_*sigC*_*-lacZ* reporter even below that of strain LMSH12 (wt P_*sigC*_*-lacZ*, *P* > 0.01). Apparently, LstR and σ^C^ jointly control expression of the *sigC* operon in inverse directions. Despite *lmo0421* overexpression in *lstR* inactivated cells, effects on the sensitivity of the lstR* or lstR* Δ*lmo0421* mutant against antibiotics affecting different steps in cell wall biosynthesis were not detected (Fig. 4c). Alterations in cell wall ultrastructure were also not observed, questioning a possible role of Lmo0421 in cell wall biosynthesis (Fig. S2).

**Figure 5:**
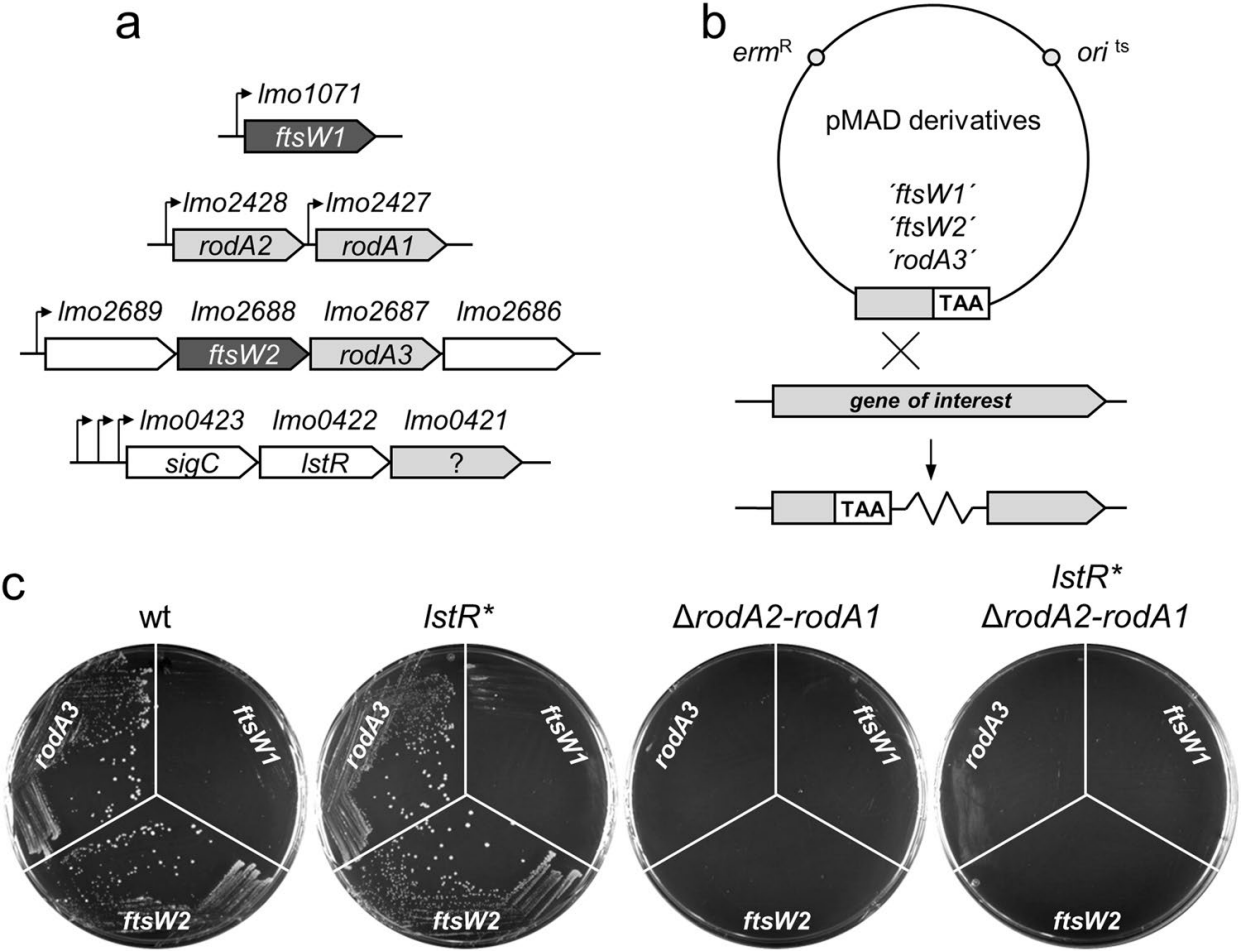
Lmo0421 cannot take over the function of any other FtsW/RodA protein. (**a**) Scheme showing genetic arrangement of the six *ftsW*/*rodA* genes in *L*. *monocytogenes*. (**b**) Scheme illustrating the way of insertional disruption chosen to inactivate the *ftsW1*, *ftsW2* and *rodA3* genes. (**c**) Insertional disruption of the *ftsW1*, *ftsW2* and *rodA3* genes in *L*. *monocytogenes* strains EGD-e (wt), LMSH39 (lstR*), LMSH67 (Δ*rodA2-rodA1*) and LMSH68 (lstR* Δ*rodA2-rodA1*). Temperature sensitive plasmids designed to disrupt the *ftsW1* (pSAH66), *ftsW2* (pSAH68), or *rodA3* genes (pSAH67) or in Campbell-type integration events were forced to integrate into their respective target gene in the different strain backgrounds during growth on BHI agar plates containing erythromycin at 42 °C. Colony formation indicates chromosomal plasmid integration and target gene disruption as depicted in panel B. Please note that all strains can grow on BHI/erythromycin plates at 30 °C.

In order to test whether Lmo0421 could substitute for FtsW1 or FtsW2, we then introduced plasmids allowing insertional disruption of *ftsW1* (*lmo1071*) and *ftsW2* (*lmo2688*) into the wild type and the lstR* mutant. These plasmids are maintained as extrachromosomal replicons at permissive temperature (30 °C) and, consequently, strains transformed with these vectors grow on erythromycin-containing BHI plates at 30 °C (data not shown). However, plasmid replication is blocked and only clones that have integrated the plasmid into the chromosome can grow in the presence of erythromycin at non-permissive temperature (42 °C, Fig. 5a,b). In good agreement with previous results^39^, *L*. *monocytogenes* wild type tolerated disruption of *ftsW2* at 42 °C, whereas *ftsW1* could not be inactivated by plasmid integration, illustrating essentiality of *ftsW1* and dispensability of *ftsW2*. Importantly, this gene essentiality pattern was not changed in the lstR* mutant, clearly demonstrating that overproduced Lmo0421 cannot compensate for the loss of FtsW1 function (Fig. 5c). In order to test whether Lmo0421 can functionally replace one of the three RodA proteins, we first deleted the *rodA2-rodA1* genes in the wild type and the lstR* mutant. These strains were then transformed with a plasmid that allows insertional disruption of *rodA3*. While *rodA3* can be readily inactivated in wild type and the lstR* mutant, where the *rodA2-rodA1* genes are still present, this is not possible in the Δ*rodA2-rodA1* strain. This result confirms previous findings showing that at least one of the three RodA proteins is required for viability of *L*. *monocytogenes*^39^. However, disruption of *rodA3* was also not possible in the lstR* Δ*rodA2-rodA1* mutant, indicating that one of the RodA homologs is required for viability of *L*. *monocytogenes* even when Lmo0421 is overexpressed. It is important to note that disruption of *ftsW2* is not tolerated in Δ*rodA2-rodA1* strains because plasmid insertion into *ftsW2* would separate *rodA3* from the promoter of the operon upstream of *lmo2689*^28^. Taken together, we conclude that Lmo0421 has neither FtsW nor RodA functionality under these conditions.

## Discussion

Shared and specific features of the four PadR-type transcriptional repressors of *L*. *monocytogenes* begin to emerge. The three repressors studied here specifically control transcription of a small set of genes, usually comprising one to two affected transcription units per repressor. Their target genes are strongly repressed under standard growth conditions in wild type and de-repressed roughly 150-fold in the respective repressor mutants. This regulation patterns suggests that they are disadvantageous during exponential growth but highly beneficial under specific conditions. Among the four, LftR is the strongest repressor as it represses its target promoters about 450-fold^21^. Another shared feature is the presence of negative feedback loops in the gene expression control circuits. LftR^21^, LltR and LstR (this work) are autoregulatory by repressing their own genes. Whether LadR represses transcription of its own gene could not be decided based on our RNA-Seq data since the monocistronic *ladR* transcript is simply absent in the Δ*ladR* mutant. However, the *ladR* and *mdrL* promoters are in close proximity^23^, so that control of both genes by a single LadR operator seems possible. These negative feedback loops switch off transcription when the inducing molecules or conditions are no longer present.

With σ^C^, a second, but positive feedback loop is enmeshed in the control circuit of the *sigC* operon. This creates the possibility to integrate a second signal so that the *sigC* operon would only be fully induced when LstR relieves its repression and σ^C^ is activated. Provided that σ^C^ is not completely active during exponential growth, this would suggest that we only observe an intermediate level of *sigC-lstR-lmo0421* transcription in the *lstR* mutant. σ^C^ is important for growth of the *L*. *monocytogenes* strain 10403 S at low^33^ and high temperatures^26^, but we could not observe similar effects in the *L*. *monocytogenes* EGD-e background (Fig. 2b and data not shown). *L*. *monocytogenes* σ^C^ shares certain homology with extracytoplasmic function (ECF) sigma factors and is the only sigma factor of this type in strain EGD-e^26^, but it is not known how exactly σ^C^ contributes to transcription of its operon. Remarkably, the *sigC* operon is not present in the entire *L*. *monocytogenes* population and only found in strains of phylogenetic lineage II^40^, to which EGD-e and 10403 S belong. Consistent with this observation and in good agreement with previous results^26,35^, σ^C^ does only activate its own promoter, so that *lmo0421* is the only target gene of σ^C^ and LstR. ECF sigma factors respond to signals that attack the integrity of the membrane and the cell wall, such as antimicrobial peptides and lytic enzymes^41^. RodA/FtsW enzymes act in peptidoglycan biosynthesis as glycosyltransferases mediating elongation of peptidoglycan chains^31,32,42^. According to our experiments, Lmo0421 cannot take over the function of any of the other five RodA/FtsW enzymes and its overproduction in an lstR* background did not affect susceptibility against moenomycin that inhibits glycosyltransferase activity in penicillin binding proteins^43^. This latter observation suggests that Lmo0421 does not contribute to peptidoglycan transglycosylation under these conditions. However, Lmo0421 may functionally replace one of the other FtsW/RodA proteins under more specific conditions, for example when the FtsW/RodA glycosyltransferases are inhibited by more specific drugs. FtsW/RodA inhibitors are not known, but just recently, a supposedly inhibitory molecule of so far unknown structure has been discovered in a natural compound library screen with a *B*. *subtilis* mutant devoid of all transglycosylase activity mediated by penicillin binding proteins^31^. This compound (preliminary designation 654/A) or related substances might be recognized by LstR (and/or σ^C^) under conditions where the house-keeping RodA/FtsW enzymes are chemically inactivated, leading to production of Lmo0421 as a 654/-resistant back-up protein. Interestingly, compound 654/A like the inductor of LftR, aurantimycin A, is produced by a soil-dwelling *Streptomyces* strain^21,31^. However, in order to test this hypothesis the identity of 654/A must be elucidated first. Alternatively, Lmo0421 might use chemically modified lipid II as substrate, which may be produced to confer resistance to antimicrobial peptides^44^. Regardless of these considerations, a role for Lmo0421 in cell wall biosynthesis is supported by a spontaneous *lmo0421* mutation found in a stable *L*. *monocytogenes* L-form strain that lacks a cell wall^45^.

An inducer for LltR is presently not known. Rhodamine 6G was found to induce *mdrL* transcription in the background of *L*. *monocytogenes* LO28 suggesting that LadR senses rhodamine 6G. However, we cannot confirm this for strain EGD-e carrying the P_*mdrL*_*-lacZ* fusion in agar diffusion test (data not shown). Moreover, rhodamine dyes are of synthetic compounds and thus, other naturally occurring LadR effector molecules must exist. Based on the above mentioned considerations we speculate that these molecules, which need to be identified in future work, might be of streptomycetes origin. This would then be another common feature of listerial PadR-like repressors.

## Materials and Methods

### Bacterial strains and growth conditions

All strains used in this study are listed in Table 2. *L*. *monocytogenes* was generally cultivated in BHI broth or on BHI agar plates at 37 °C if not stated otherwise. Where required, antibiotics and supplements were added at the following concentrations: erythromycin (5 µg mL^−1^), kanamycin (50 µg mL^−1^) and X-Gal (100 µg mL^−1^). *Escherichia coli* TOP10 was used as standard cloning host^46^.

**Table 2:** Strains and plasmids used in this study.

name	relevant characteristics	source*/reference
**plasmids**		
pIMK3	P_*help*_*-lacO lacI neo*	^47^
pMAD	*bla erm bgaB*	^49^
pBP117	*lacZ neo*	^21^
pSAH1	*bla erm bgaB* Δ*ladR*	this work
pSAH3	*bla erm bgaB* Δ*lstR*	this work
pSAH4	*bla erm bgaB lltR (lmo0599)*	this work
pSAH5	*bla erm bgaB lltR* ^*L49A R51A L52A*^	this work
pSAH12	P_*sigC*_*-lacZ neo*	this work
pSAH14	P_*mdrL*_*-lacZ neo*	this work
pSAH15	P_*lltR*_*-lacZ neo*	this work
pSAH32	*bla erm bgaB* Δ*lmo0421*	this work
pSAH33	*bla erm bgaB lstR* ^*L90A L92A L93A*^	this work
pSAH34	*bla erm bgaB lstR*^*L90A L92A L93A*^ Δ*lmo0421*	this work
pSAH37	P_*help*_*-lacO-lltR lacI neo*	this work
pSAH45	*bla erm bgaB lltR*^*L49A R51A L52A*^ Δ*lmo0600*	this work
pSAH46	*bla erm bgaB lltR*^*L49A R51A L52A*^ Δ*lmo0601*	this work
pSAH62	*bla erm bgaB* Δ*lmo2428-2427*	this work
pSAH66	*bla erm bgaB ‘ftsW1’*	this work
pSAH67	*bla erm bgaB ‘rodA3’*	this work
pSAH68	*bla erm bgaB ‘ftsW2’*	this work
pSAH69	*bla erm bgaB* sigC*	this work
***L***. ***monocytogenes*** **strains**		
EGD-e	wild type, serovar 1/2a strain	lab collection
LMSH16	*attB::lacZ neo*	^21^
LMSH1	Δ*ladR* (*lmo1408*)	pSAH1 ↔ EGD-e
LMSH2	Δ*lstR* (*lmo0422*)	pSAH3 ↔ EGD-e
LMSH3	*lltR* ^*L49A R51A L52A*^	pSAH5 ↔ EGD-e
LMSH10	*attB::*P_*mdrL*_*-lacZ neo*	pSAH14 → EGD-e
LMSH11	Δ*ladR attB::*P_*mdrL*_*-lacZ neo*	pSAH14 → LMSH1
LMSH12	*attB::*P_*sigC*_*-lacZ neo*	pSAH12 → EGD-e
LMSH13	Δ*lstR attB::*P_*sigC*_*-lacZ neo*	pSAH12 → LMSH2
LMSH14	*attB::*P_*lltR*_*-lacZ neo*	pSAH15 → EGD-e
LMSH15	*lltR*^*L49A R51A L52A*^ *attB::*P_*lltR*_*-lacZ neo*	pSAH15 → LMSH3
LMSH39	*lstR* ^*L90A L92A L93A*^	pSAH33 ↔ EGD-e
LMSH40	*lstR*^*L90A L92A L93A*^ Δ*lmo0421*	pSAH34 ↔ LMSH39
LMSH42	*lltR*^*L49A R51A L52A*^ *attB::*P_*help*_*-lacO-lltR lacI neo*	pSAH37 → LMSH3
LMSH50	*lltR*^*L49A R51A L52A*^ Δ*lmo0600*	pSAH45 ↔ LMSH3
LMSH51	*lltR*^*L49A R51A L52A*^ Δ*lmo0601*	pSAH46 ↔ LMSH3
LMSH63	*lstR*^*L90A L92A L93A*^ *attB::*P_*sigC*_*-lacZ neo*	pSAH12 → LMSH39
LMSH67	Δ*lmo2428-2427*	pSAH62 ↔ EGD-e
LMSH68	*lstR*^*L90A L92A L93A*^ Δ*lmo2428-2427*	pSAH62 ↔ LMSH39
LMSH89	sigC* (*lmo0423*)	pSAH69 ↔ EGD-e
LMSH90	*lstR*^*L90A L92A L93A*^ sigC*	pSAH69 ↔ LMSH39
LMSH96	sigC* attB::P_*sigC*_*-lacZ neo*	pSAH12 → LMSH89
LMSH97	*lstR*^*L90A L92A L93A*^ sigC* attB::P_*sigC*_*-lacZ neo*	pSAH12 → LMSH90

^*^The arrow (→) stands for a transformation event and the double arrow (↔) indicates gene deletions obtained by chromosomal insertion and subsequent excision of pMAD plasmid derivatives (see experimental procedures for details).

### General methods, manipulation of DNA and oligonucleotide primers

Transformation of *E*. *coli* and isolation of plasmid DNA was performed according to standard methods^46^. Preparation of electro-competent *L*. *monocytogenes* cells and transformation of *L*. *monocytogenes* were done as described elsewhere^47^. Restriction and ligation of DNA was carried out as detailed in the manufacturer’s instructions. For restriction free modification of plasmids an altered version of the original QuikChange mutagenesis protocol was employed^48^. All primer sequences are listed in Table 3. Antibiotic susceptibility assays were recorded using filter discs soaked with solutions of antibiotics as indicated. *L*. *monocytogenes* colonies were grown over night in BHI broth and used to swab-inoculate BHI agar plates. Filter discs soaked with antibiotics were placed on top of the agar surface and the plates were incubated at 37 °C overnight.

**Table 3:** Oligonucleotides used in this study.

name	sequence (5′ → 3′)
SAH32	CAGATCTATCGATGCATGCCATGGAGGAAAGGAAGAGGAGAATTATG
SAH33	AATTCAGAGGTGCTATTGTGTCGACAAAAACCGGCGAACTTAATATTCGC
SAH34	ATTAAGTTCGCCGGTTTTTGTCGACACAATAGCACCTCTGAATTTC
SAH35	CCTCGCGTCGGGCGATATCGGATCCGGCCGATATTTGAACAAATGG
SAH36	CTCGCGTCGGGCGATATCGGATCCCAGGGAGATAGCTACTAGGG
SAH37	AGGAGGTTTAATCGTCGACATGAGTTCTTCTACATTTGAAG
SAH38	TGTAGAAGAACTCATGTCGACGATTAAACCTCCTTTTTCATCTTATTC
SAH39	CAGATCTATCGATGCATGCCATGGGTTAATCATGGTGGGCGTCG
SAH52	GATCTATCGATGCATGCCATGGATGGAGGTTAACCCGCAGTTC
SAH53	GCTTCTAGAATTCGAGCTCCCTTATTCATTTACTGCTTCCCCCTC
SAH58	CCGGTAGCAAGAGCAGCAGTAAAAGAAGAGTACTGTTC
SAH59	TTTTACTGCTGCTCTTGCTACCGGATAAATAGCACCTTC
SAH109	CTAGAACTAGTGGATCCCGCATGTTCTTAGCGACTGC
SAH110	GTAAAACGACGGGGAATTCCATTTAAGTTTCACCTTCTTCTGC
SAH113	CTAGAACTAGTGGATCCCTATCTTGGGAATGCTTATGAAC
SAH114	GTAAAACGACGGGGAATTCCATAATACAACTACACTTCCC
SAH115	CTAGAACTAGTGGATCCCTCACGCATAAACTTATCTCTCC
SAH116	CGACGGGGAATTCCTGCAGCATGCTAACTCCTCCATTCTG
SAH175	CTATCGATGCATGCCATGGTCCATCTTCCTCCTCCAG
SAH176	GCACATGCAGCTGAAAACAAGAAAATCCTATC
SAH177	AGCTGCATGTGCCAGAATATAAAGCTGACC
SAH178	CTGCAGAAGCTTCTAGAATTCGACAGCGGAAGATTTAACG
SAH179	CTATCGATGCATGCCATGGTGGCAAGGATGAGGAGC
SAH180	CTTTATGAGTTAATTTAGTGTATAACCGACATG
SAH181	CACTAAATTAACTCATAAAGAAGCCTCCTC
SAH185	CCCATGGAAAAGGATCCATGGAGGTTAACCCGCAGTTC
SAH186	CGAATTCCTGCAGCCCGGGTTATTCATTTACTGCTTCCCCCTC
SAH212	CTATCGATGCATGCCATGGATGGAGGTTAACCCGCAGTTC
SAH213	AGTGATGTTTATTCATTTACTGCTTCCCCCT
SAH214	TGAATAAACATCACTTAAGCAAAAAACT
SAH215	CTGCAGAAGCTTCTAGAATTCTCATCTTCTTCAGGCACCGT
SAH216	CTATCGATGCATGCCATGGTGACCTTGGTAAGCCAGAAG
SAH217	TTCGATTTTATGCATTTTTCCCGCCTCC
SAH218	TGCATAAAATCGAAAAATAAAAAATCAGTGCGCT
SAH219	CTGCAGAAGCTTCTAGAATTCAGCTCCGTGCCAAGTCC
SAH253	CTATCGATGCATGCCATGGTCTCTGGCTTGTTTTAACC
SAH254	TTAGGTACCGAATCGAACGACAGCGGAAG
SAH255	TTCGGTACCTAAGAAAAAACTAGATGCAGTAC
SAH256	CTGCAGAAGCTTCTAGAATTCTGCAGAAGAAGGTGAAAC
SAH257	CTATCGATGCATGCCATGGTCGTGCTATGTTTATTTGGGC
SAH258	CTGCAGAAGCTTCTAGAATTCTTATTAACCCATACCAATCCCA
SAH259	CTATCGATGCATGCCATGGTCTGCTTTCACCCATTC
SAH260	ATGGCCTCTTAAAATGAAAATACAGAAGTC
SAH261	CATTTTAAGAGGCCATTGTTTTCCGTC
SAH262	CTGCAGAAGCTTCTAGAATTCTGATTTAGTGGCGTATGG
SAH263	CTATCGATGCATGCCATGGTTATTACCCACCGCTCCCAATC
SAH264	CTGCAGAAGCTTCTAGAATTCACGAGTTATGGTGTTGCTG
SAH269	CTGCAGAAGCTTCTAGAATTCCCGGATTATTTTATCGGTGTTC
SAH270	CTATCGATGCATGCCATGGTTATTATCCCCGAACCAACAGC

### Construction of plasmids and strains

Plasmid pSAH1 was constructed for deletion of the *ladR* gene. To this end, fragments up- and downstream of *ladR* were amplified by PCR using the primers SAH32/SAH33 and SAH34/SAH35 and both fragments were fused together by splicing by overlapping extension PCR (SOE-PCR) with SAH32/SAH35 as the primers. The resulting fragment was ligated into pMAD using BamHI/EcoRI.

Plasmid pSAH3 was constructed for removal of *lstR*. To this end, regions up- and downstream of *lstR* were amplified with the primers SAH038/SAH039 and SAH036/SAH037, respectively. These fragments were fused together by SOE-PCR and the resulting fragment was cloned into pMAD using BamHI/NcoI. An unwanted duplication of around 50 bp directly after the BamHI restriction site was removed by digesting the plasmid with BamHI and subsequent self-ligation, finally yielding pSAH3.

Plasmid pSAH5 was constructed for the introduction of the *lltR*^*L49A*^ ^*R51A*^ ^*L52A*^ triple mutation (lltR*) into the chromosome and was obtained in two steps. First, the *lltR* gene was amplified by PCR from EGD-e chromosomal DNA using the primer pair SAH52/SAH53 and cloned into pMAD using EcoRI/NcoI, resulting in plasmid pSAH4. The *L49A R51A L52A* exchanges were then introduced into pSAH4 by quikchange mutagenesis using SAH58/SAH59 as the mutagenic primers.

Plasmid pSAH33 was generated to introduce the *lstR*^*L90A L92A L93A*^ (lstR*) into the chromosome. To this end, fragments upstream and downstream of the region to be mutated were amplified with primers SAH178/SAH177 and SAH176/SAH175 (SAH177 and SAH176 introduced the lstR* mutations), both fragments were combined by SOE-PCR and the resulting fragment was introduced into pMAD by restriction-free cloning.

Plasmid pSAH32 facilitates *lmo0421* deletion and was obtained by amplification of fragments up- and downstream of *lmo0421* using the primers SAH178/SAH181 and SAH180/SAH179, respectively. Both fragments were fused together in a SOE-PCR and the resulting fragment was cloned into pMAD using EcoRI/NcoI. Plasmid pSAH32 was then used as the template in a quick change PCR using the primers SAH176/SAH177 to introduced the lstR* mutation, yielding pSAH34.

In order to remove *lmo0600* from lltR* cells, plasmid pSAH45 was constructed. To this end, fragments up- and downstream of *lmo0600* were amplified in PCRs with SAH212/SAH213 and SAH214/SAH215 and chromosomal DNA of strain LMSH3 (lltR*) as the template, respectively, and joined in a SOE-PCR, the product of which was inserted into pMAD by restriction-free cloning.

Plasmid pSAH46 was generated to remove the *lmo0601* gene. Here, *lmo0601* up- and downstream fragments were PCR amplified with SAH216/SAH217 and SAH218/SAH219, respectively, joined by SOE-PCR and inserted into pMAD by restriction-free cloning.

Plasmid pSAH62 was designed for removal of the *rodA2-rodA1* genes. For this, fragments up- and downstream to the *rodA2-rodA1* cluster were amplified with SAH262/SAH261 and SAH260/SAH259, spliced together by SOE-PCR and introduced into pMAD by restriction-free cloning.

Plasmid pSAH69 was constructed by amplification of the 5′- and 3′-halves of *sigC* using the primer pairs SAH256/SAH255 and SAH254/SAH253, respectively, their subsequent joining by SOE-PCR and the cloning of the obtained fragment into pMAD using restriction-free cloning. SAH255 and SAH254 introduced a premature stop codon at the 39^th^ base pair triplet of *sigC* followed by a KpnI site (sigC*).

Derivatives of pMAD designed for gene deletions were transformed into the respective *L*. *monocytogenes* recipient strains and genes were deleted as described elsewhere^49^. All gene deletions were confirmed by PCR.

For insertional disruption of *ftsW1*, *rodA3* and *ftsW2*, plasmids pSAH66, pSAH67 and pSAH68, respectively, were constructed. To this end, internal gene fragments were amplified by PCR using primers SAH257/SAH258 (*ftsW1*), SAH269/SAH270 (*rodA3*) and SAH263/SAH264 (*ftsW2*) and inserted into pMAD by restriction-free cloning. Plasmids pSAH66-67 were then introduced into *L*. *monocytogenes* strains by electroporation and transformants were selected on BHI agar containing erythromycin at 30 °C.

For construction of promoter-*lacZ* fusions, fragments carrying the P_*sigC*_, P_*mdrL*_ and P_*lltR*_ promoters were amplified by PCR using the primer pairs SAH109/SAH110, SAH113/SAH114 and SAH115/SAH116, respectively, and introduced into pBP117 by restriction-free cloning^50^, resulting in plasmids pSAH12, pSAH14 and pSAH15, respectively.

Plasmid pSAH37 for IPTG-dependent expression of *lltR* was generated by amplification of *lltR* using primers SAH185/SAH186 and the insertion of the resulting fragment into pIMK3 by restriction-free cloning.

Derivatives of pBP117 and pIMK3 were introduced into *L*. *monocytogenes* strains by electroporation and selected on BHI agar plates containing kanamycin. Plasmid insertion at the *attB* site of the tRNA^Arg^ locus was verified by PCR.

### mRNA isolation

mRNA was isolated as described previously^21^. Briefly, cells from 25 ml of a culture grown in BHI broth (OD_600_ of ~0.8) was mixed with 25 ml ice cold killing buffer (20 mM Tris-HCl pH 7.5, 5 mM MgCl_2_, 20 mM NaN_3_) and harvested by centrifugation after 5 min incubation on ice.

RNA extraction followed the protocol of Gertz *et al*.^51^ with modifications as described^18^. 10 µg total RNA were digested with DNAse using the RNase-Free DNase Set (Qiagen) and then purified using RNA clean & concentrator columns (Zymo Research) for purification for RNA molecules longer than 200 nucleotides. RNA quality was assessed using Agilent Bioanalyzer RNA Nano chips. rRNA was depleted using the Ribo-Zero Bacteria Kit (Illumina), 2 µg purified RNA was treated with 10 µl Ribo-ZeroRemoval Solution and pelleted by ethanol precipitation. RNA concentrations were determined in a Qubit® fluorometer.

### Library preparation and sequencing

RNA libraries were prepared using the TruSeq® Stranded mRNA Kit as described^35^. RNA transcripts were quantified by quasi-mapping of the reads to the *L*. *monocytogenes* EGD-e cDNA (Listeria_monocytogenes_egd_e.ASM19603v1.cds.all.fa.gz), provided by the Ensembl Genomes server^52^, using the Salmon software^53^. Average expression from three biological replicates of the mutant divided by the average expression from three biological replicates of the wildtype gave the differential expression ratio. Log2-transformed transcript counts from three biological replicates were then used to calculate *P* values using Students *t*-test. Significantly differentially expressed genes were defined as having a *P*-value less than 0.01, an absolute differential expression factor of more than 2 and an expression level of at least 10 TPM.

### β-galactosidase reporter assays

Reporter strain cultures were grown in BHI broth at 37 °C until an OD_600_ of 0.5–0.6. Cells were pelleted, washed once with 500 µl H_2_O and then resuspended in 1.2 ml Z-Buffer (60 mM Na_2_HPO_4_, 40 mM NaH_2_PO_4_, 10 mM KCl, 1 mM MgSO_4_, 20 mM 2-mercaptoethanol). Cells were lysed by sonification and cellular debris was removed by centrifugation. Protein content was determined using Roti®-Nanoquant. Samples were diluted in Z buffer to a final volume of 1 ml and incubated at 30 °C for 10 minutes. The reaction was started by addition of 200 µl ONPG solution (4 mg/ml in Z-Buffer) and stopped by addition of 500 µl 1 M Na_2_CO_3_ as soon as the first sample turned clearly yellow. Absorption was measured at 420 nm and Miller units (MU) were calculated.

### Infection experiments

Experimental infections were carried out as described earlier^54^. Briefly, 3 × 10^5^ J774.A1 mouse ascites macrophages (ATCC) were seeded into the wells of a 24 multi well plate and cultivated in 1 ml high glucose DMEM medium (4.5 g/l glucose, 110 mg/l sodium pyruvate, 584 mg/l L-glutamine) supplemented with 10% fetal calf serum (FCS) for one day at 37 °C in a 5% CO_2_ atmosphere. 5 × 10^4^ bacteria from overnight cultures were resuspended in 1 ml of fresh DMEM without FCS and this inoculum was used to infect the J774 cells during an incubation step of 1 h at 37 °C. Next, the wells were washed once with PBS and all extracellular bacteria were killed during another 1 h incubation step in DMEM (without FCS) containing 40 µg/ml gentamicin. The wells were covered with fresh DMEM (without FCS) containing 10 µg/ml gentamicin after one more PBS wash step, and then incubated at 37 °C in a 5% CO_2_ atmosphere. Sampling was performed at various time points by lysing the cells in 1 ml of ice-cold PBS containing 0.1% Triton X-100. Serial dilutions were plated on BHI agar plates in order to count the recovered bacterial colonies.

### Electron microscopy

Scanning electron microscopy and ultrathin section transmission electron microscopy were performed essentially as described earlier^55^.

## Supplementary information

Supplementary Figures S1 and S2

## Data Availability

RNA sequencing raw files are available at the NCBI Geo Server (https://www.ncbi.nlm.nih.gov/geo/) under study accession numbers: GSE129904 (*ladR*), GSE129909 (*lstR*) and GSE129910 (*lltR*).
