# Supplementary material for: PadR-type repressors controlling production of a non-canonical FtsW/RodA homologue and other trans-membrane proteins
Source: Sci Rep. 2019 Jul 11;9:10023. doi: 10.1038/s41598-019-46347-w (PMC6624303; doi:10.1038/s41598-019-46347-w)
Supplement: Supplementary file 1 — Supplementary Figures S1 and S2 [file 41598_2019_46347_MOESM1_ESM.pdf]

Supplementary Information to:

PadR-type repressors controlling production of a non-canonical FtsW/RodA homologue and other trans-membrane proteins

Samuel Hauf[1], Lars Möller[2], Stephan Fuchs[3], and Sven Halbedel[1,*]

[1] FG11 Division of Enteropathogenic bacteria and *Legionella*, Robert Koch Institute, Burgstrasse 37, 38855 Wernigerode, Germany;

[2] ZBS 4 - Advanced Light and Electron Microscopy, Robert Koch Institute, Nordufer 20, 13353 Berlin, Germany;

[3] FG13 Nosocomial Pathogens and Antibiotic Resistances, Robert Koch Institute, Burgstrasse 37, 38855 Wernigerode, Germany;

[*] Corresponding author:

halbedels@rki.de, Robert Koch Institute, FG11 Division of Enteropathogenic bacteria and *Legionella*, Burgstrasse 37, D-38855 Wernigerode, Germany; phone: +49-30-18754-4323; fax: +49-30-18754-4207;

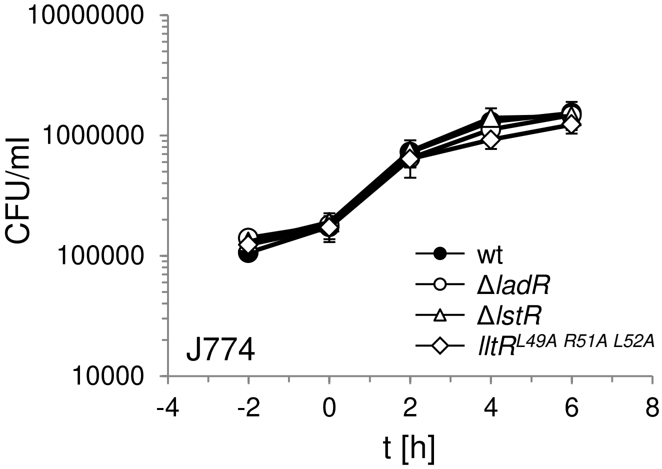

Figure S1: Intracellular growth of *L. monocytogenes ladR*, *lstR* and *lltR* mutants.

L. monocytogenes strains EGD-e (wt), LMSH1 (Δ*ladR*), LMSH2 (Δ*lstR*) and LMSH3 (*lltR*$^{L49A\ R51A\ L52A}$) were used to infect a confluent layer of J774 mouse macrophages and intracellular multiplication was determined over time. Average values and standard deviations were calculated from an experiment performed in triplicate.

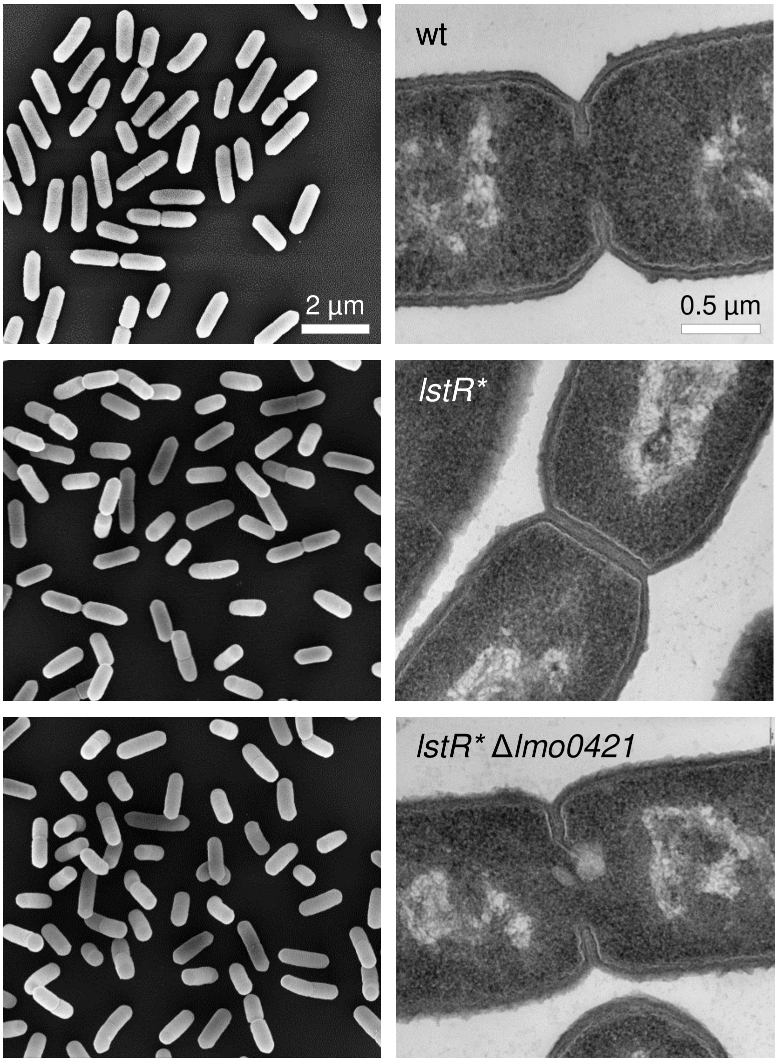

Figure S2: Morphology of *L. monocytogenes* mutants lacking *lstR* and *lmo0421*.
Scanning electron microscopy (left) and transmission electron microscopy of ultrathin sections (right) of fixed whole cells of *L. monocytogenes* strains EGD-e (wt), LMSH39 (*lstR*)* and LMSH40 (*lstR* Δ*lmo0421*) grown in BHI broth at 37°C to mid-exponential growth phase.
